# Human herpesvirus 6 major immediate early promoter has strong activity in T cells and is useful for heterologous gene expression

**DOI:** 10.1186/1743-422X-8-9

**Published:** 2011-01-11

**Authors:** Masaaki Matsuura, Masaya Takemoto, Koichi Yamanishi, Yasuko Mori

**Affiliations:** 1 Laboratoy of Virology, Division of Biomedical Research, National Institute of Biomedical Innovation, 7-6-8, Saito-Asagi, Ibaraki, Osaka 567-0085, Japan; 2 Kanonji Institute, the Research Foundation for Microbial Diseases of Osaka University, 2-9-41, ahata-cho, Kanonji, Kagawa, 768-0061, Japan; 3 Division of Clinical Virology, Kobe University Graduate School of Medicine, 7-5-1, Kusunoki-cho, Chuo-ku, Kobe, 650-0017, Japan

## Abstract

**Background:**

Human herpesvirus-6 (HHV-6) is a beta-herpesvirus. HHV-6 infects and replicates in T cells. The HHV-6-encoded major immediate early gene (MIE) is expressed at the immediate-early infection phase. Human cytomegalovirus major immediate early promoter (CMV MIEp) is commercially available for the expression of various heterologous genes. Here we identified the HHV-6 MIE promoter (MIEp) and compared its activity with that of CMV MIEp in various cell lines.

**Methods:**

The HHV-6 MIEp and some HHV-6 MIEp variants were amplified by PCR from HHV-6B strain HST. These fragments and CMV MIEp were subcloned into the pGL-3 luciferase reporter plasmid and subjected to luciferase reporter assay. In addition, to investigate whether the HHV-6 MIEp could be used as the promoter for expression of foreign genes in a recombinant varicella-zoster virus, we inserted HHV-6 MIEp-DsRed expression casette into the varicella-zoster virus genome.

**Results:**

HHV-6 MIEp showed strong activity in T cells compared with CMV MIEp, and the presence of intron 1 of the MIE gene increased its activity. The NF-κB-binding site, which lies within the R3 repeat, was critical for this activity. Moreover, the HHV-6 MIEp drove heterologous gene expression in recombinant varicella-zoster virus-infected cells.

**Conclusions:**

These data suggest that HHV-6 MIEp functions more strongly than CMV MIEp in various T-cell lines.

## Background

Human herpesvirus 6 (HHV-6) was first isolated in 1986 from the peripheral blood of patients with lymphoproliferative disorders and AIDS [1,2]. The virus was subsequently shown to be ubiquitous in healthy adults [3]. HHV-6 has been isolated from infants with exanthema subitum, a common childhood disease [4]. Later, HHV-6 isolates were classified into two variants, A and B (HHV-6A and HHV-6B), based on molecular and biological criteria [5-8]. HHV-6B causes exanthema subitum [4], while the pathogenesis of HHV-6A is still unknown. HHV-6 has the unique feature of being able to replicate and produce progeny in T cells [9,10]. The HHV-6 genome is a double-stranded DNA of approximately 160 kbp, consisting of a unique long region of 140 kbp flanked by 10-kbp direct repeats, and there is 90% identity between the two variants [11].

HHV-6 belongs to the beta-herpesvirus subfamily, which includes human cytomegalovirus (HCMV) and human herpesvirus 7 (HHV-7) [12]. The betaherpesviruses have extensive domains of similar genomic organization, with conserved herpesvirus gene blocks in the unique region of their genome [13]. HCMV's major immediate early (MIE) enhancer-containing promoter has been developed [14,15]; it is currently commercially available and is used to drive the expression of various genes. The MIE promoter controls the expression of two IE transcripts, designated IE1 (UL123) and IE2 (UL122) [16]. HHV-6 has positional homologs of UL123 and UL122; they are U89 and U86, which are designated IE1 and IE2, respectively [11,13,17,18]. The HHV-6 IE1 and IE2 transcripts are formed by alternative splicing [19,20]. Recently Takemoto et al. reported that the R3 region in the right end of HHV-6 is a strong enhancer of another HHV-6 immediate early gene, U95 [21]. R3 is positioned between U95 and U89; therefore, the region containing R3 is predicted to also contain promoter activity for the IE1 and IE2 genes. In other words, this location is predicted to be a positional homolog of the HCMV MIE promoter.

In this study, we identified the promoter region that regulates the HHV-6 MIE gene, and analyzed its activity. As expected, part of the R3 region was critical for the promoter activity. We also found that the first intron encoded by the IE1 gene enhanced HHV-6 MIE promoter (HHV-6 MIEp) activity, and that HHV-6 MIEp with the first intron had significantly stronger activity than the HCMV MIE promoter, especially in T-cell lines. The HHV-6 MIEp was able to express heterologous genes in a recombinant varicella-zoster virus, indicating that it could be useful for expressing various genes in a similar manner as the CMV MIE promoter.

## Results

### The HHV-6 major immediate-early promoter had stronger activity than the CMV promoter in T-cell lines

**Figure 1:**
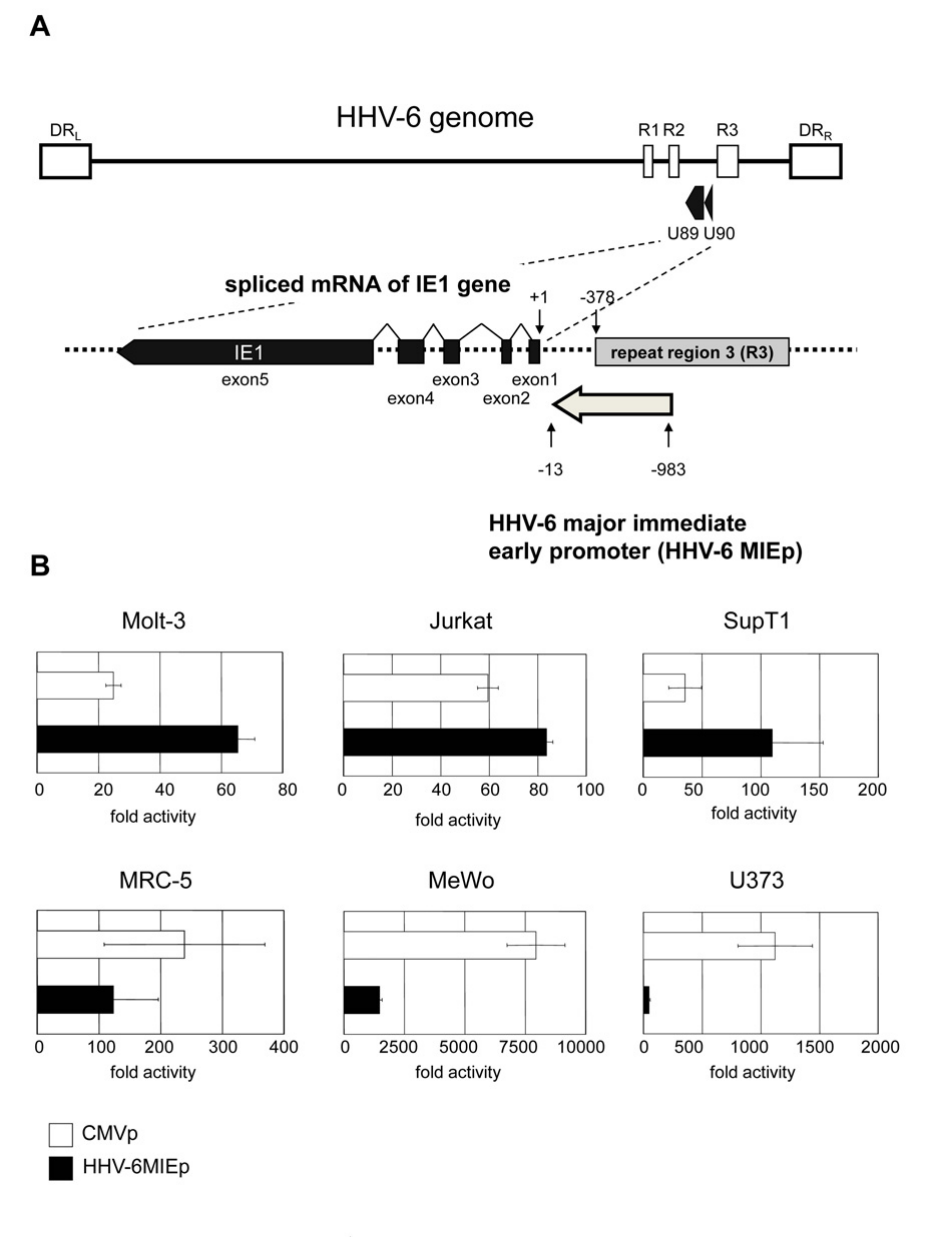
**Comparison of the activities of the HHV-6 MIE promoter and CMV promoter**. (A) The HHV-6 genome is a double-stranded DNA molecule of approximately 160 kbp, and is composed of a single long unique sequence (U) flanked by identical direct repeats (DR_L _and DR_R_). IE1 maps to open reading frames U90 and U89. The HHV-6 MIE promoter of 971 bp, located upstream of exon 1 of IE1, was amplified by PCR from the HHV-6B strain HST genome. Bases are numbered starting with the transcriptional start site for the IE1 gene. (B) The HHV-6 MIE promoter and CMV promoter were subcloned into the pGL3-basic plasmid, and the resultant plasmids were transfected into various cell lines (Jurkat, Molt-3, SupT1, MRC-5, MeWo, and U373 cells). At 24-hr post-transfection, the cells were harvested and subjected to the luciferase activity assay. The mean fold-activity relative to that of blank pGL3-basic plasmid-transfected cells and standard deviation for three independent experiments were plotted.

The 5' end of the mRNA encoded by the HHV-6 immediate early 1 (IE1) gene is located at base 139442 of the HHV-6 strain HST genome [11,22]. The 971-bp region upstream of the IE1 gene, including the R3 repeat, was suspected to include the HHV-6 major immediate-early promoter (HHV-6MIEp). The promoter region used in this study is illustrated in Figure 1A. First, to investigate the relative strength of the HHV-6 MIE promoter in various cell types, reporter gene assays were performed using the luciferase gene expression system. A plasmid containing the luciferase gene under the HHV-6MIEp was transfected into MRC-5, MeWo, U373, Molt-3, SupT1, and Jurkat cells. The pRL-TK plasmid, encoding Renilla luciferase under the transcriptional control of the herpes simplex virus thymidine kinase (HSV-TK) promoter, was co-transfected to normalize the transfection efficiency. The data show the fold-increase relative to the value of cells transfected with a blank plasmid, pGL3-basic (Promega). As shown in Figure 1B, the activity of the HHV-6 MIE promoter was weaker than that of the CMV promoter (CMV MIEp) in MRC-5, U373 and Mewo cells, while the activity was stronger than that of the CMV promoter in Molt-3, SupT1, and Jurkat cells.

**Figure 2:**
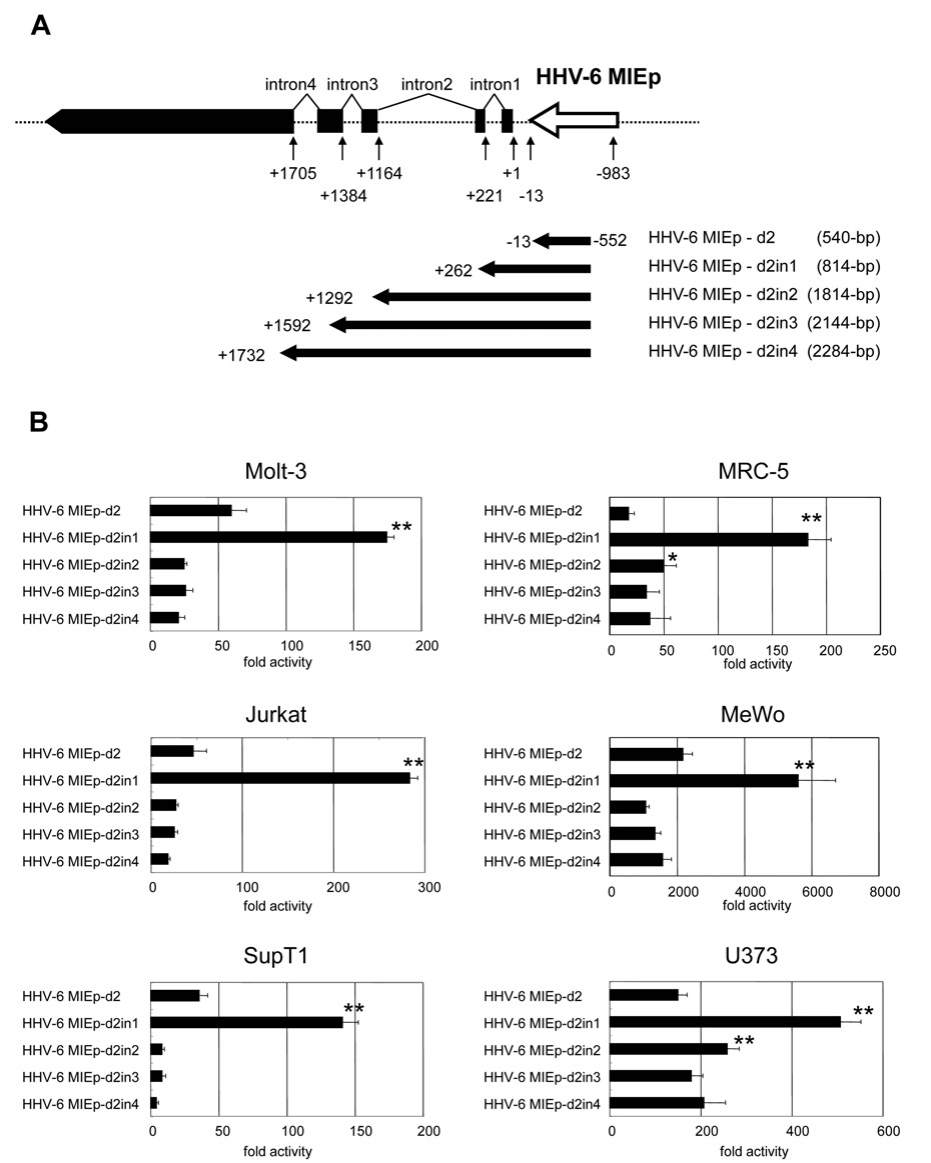
**The activity of the HHV-6 MIE promoter including intron(s) of the IE gene**. Schematic representation of the HHV-6 MIE promoter variants, including introns 1-4 of the IE1 gene, used for analysis of the introns. The numbers indicate the nucleotide position from the 5' end of mRNA encoded by the IE1 gene. The +1 indicates the 5' end. The name and size of each promoter used here are shown at the right. (B) Luciferase assays were performed after transfection in various cell lines (Molt-3, Jurkat, SupT-1, MRC-5, Mewo, and U373). The mean fold-activity of each HHV-6 MIE promoter variant relative to that of the blank pGL3-basic plasmid is shown by the horizontal bar. Standard deviation for three independent experiments is indicated. One asterisk indicates that the P value is < 0.05 and two asterisks indicate that the P value is < 0.01 in comparison with HHV-6MIEp-d2 (without intron 1), as determined by Student's unpaired two-tailed t-test.

The mRNAs encoded by the HHV-6 IE1 gene are produced by alternative splicing (Figure 1A). It is known that introns within some genes can elevate the protein expression level by either enhancing the promoter activity or stabilizing the mRNA [23]. In HCMV, the addition of intron A from the IE1 gene to the IE promoter/enhancer increases the promoter activity [24]. Therefore, we examined the role of the introns encoded by the HHV-6 MIE genes in the HHV-6 MIE promoter activity. To examine this, several HHV-6 MIE promoter variants containing introns 1-4 were constructed (Figure 2A), and the activities were compared by performing the reporter assay in various cells. As shown in Figure 2B, in the presence of intron 1, the promoter activity was significantly upregulated in all the cells compared to the HHV-6 MIE promoter without intron 1. In contrast, the further addition of introns 1-2, 1-3, or 1-4 downregulated the promoter activity (Figure 2B). Therefore the HHV-6 MIE promoter containing intron 1 (HHV-6MIEp-in1), whose length is 1245-bp, was included in the remaining experiments.

**Figure 3:**
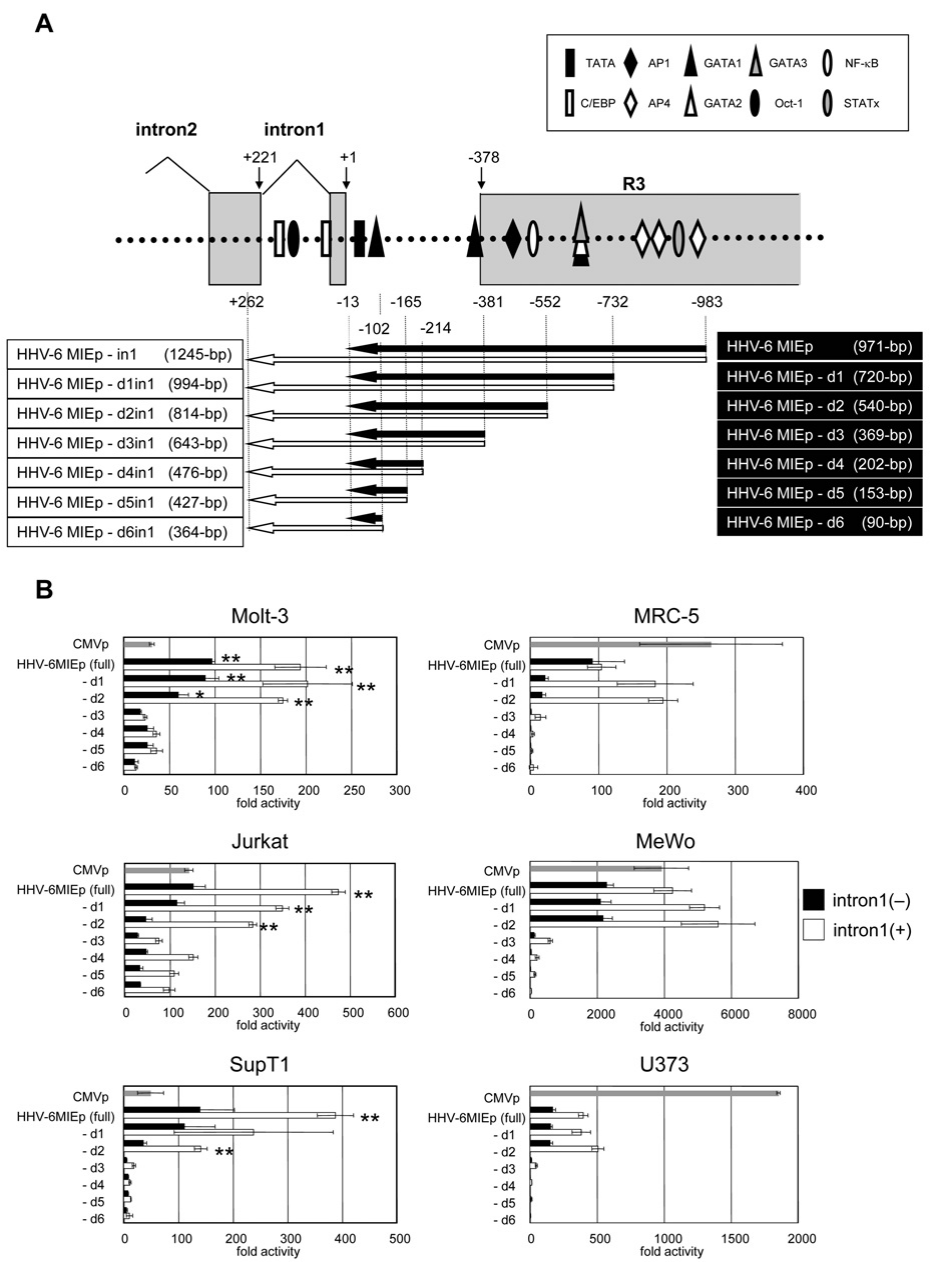
**Comparison of promoter activity in deletion mutants of HHV-6MIEp and HHV-6MIEp-in1**. (A) Schematic representation of 5'-deletion mutants of the HHV-6MIEp (black arrows) and of the HHV-6MIEp-in1, which is the HHV-6MIEp including intron 1 (white arrows). The name and size of each promoter are shown at the right (without intron 1) and left (with intron 1). Putative transcription factor-binding sites, predicted by TFSEARCH (http://www.cbrc.jp/research/db/TFSEARCH.html), are shown. (B) Luciferase assays were performed in various cell lines. The mean fold-activity relative to that of the blank pGL3-basic plasmid is indicated by the horizontal bars. The standard deviation for three independent experiments is indicated. The activities of CMV MIEp, deletion mutants of the HHV-6MIEp, and deletion mutants of the HHV-6MIEp-in1, are indicated by gray, black, and white bars, respectively. One asterisk indicates that the P value is < 0.05 and two asterisks indicate that the P value is < 0.01 in comparison with the CMV MIEp, as determined by Student's unpaired two-tailed t-test.

Next, to determine the region that contributes to the promoter activity, various deletion mutants of both HHV6MIEp and HHV6MIEp-in1 were constructed (Figure 3A), and their activities were examined and compared by reporter assays in various cell lines. As shown in Figure 3B, the HHV-6MIEp-d3 promoter activity decreased compared to that of HHV-6MIEp-d2 (both with and without intron 1), showing that the region at nt positions from -381 to -552, which lies within R3, is important for the activity. In addition, the activities of HHV-6MIEp and HHV-6MIEp-in1 were significantly stronger than CMV MIEp activity in Jurkat, Motl-3, and SupT1 cells, suggesting that the HHV-6 MIE promoter has higher activity than the CMV promoter in certain cells, especially in T cells. This property of the HHV-6 MIE promoter might render it as a promising candidate for efficient protein expression in T cells.

**Figure 4:**
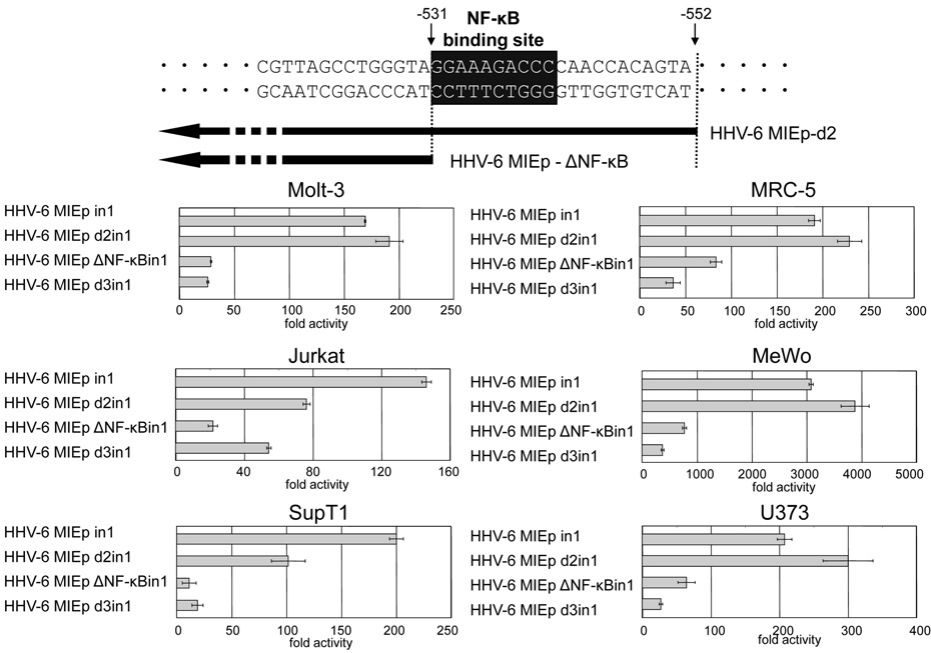
**The NF-kB-binding site is critical for the promoter activity of 6MIEp**. (A) To investigate the importance of the NF-κB-binding site for the promoter activity of 6MIEp, a 5'-deletion mutant of the 6MIEp lacking the NF-κB-binding site (white letters in black box) was constructed. (B) The Luciferase assay was performed in various cell lines. The mean fold-activity relative to that of the blank pGL3-basic plasmid is indicated by the horizontal bars. The standard deviation for three independent experiments is indicated.

The region at nt positions -381 to -552, which lies within R3, is predicted to have an NF-κB-binding site and AP-1-binding site (Figure 3A). Takemoto et al. reported that the NF-κB-binding site in the R3 region plays an important role in U95 promoter activity [21]. We hypothesized that the NF-κB-binding site plays a major role in the HHV-6MIEp promoter activity as well. To investigate this, we constructed a promoter in which the NF-κB-binding site was deleted (HHV-6MIEpΔNF-κBin1) (Figure 4), and examined its activity in various cell lines. As shown in Figure 4, the NF-κB-binding site-deleted promoter HHV-6MIEpΔNF-κBin1 exhibited significantly decreased promoter activity in all cell lines, indicating that the NF-κB-binding site in the HHV-6MIEp plays an important role in its promoter activity.

### The HHV-6 MIE promoter could drive the expression of foreign gene in a recombinant varicella virus

We recently constructed a recombinant varicella vaccine Oka strain (vOka) expressing the MuV (mumps virus) HN (hemaglutinin-neuraminidase) gene, as a possible candidate for a polyvalent vaccine for both varicella zoster virus (VZV) and MuV infections [25]. In that study, the CMV promoter was used to control the HN gene. Since the HHV-6 MIE promoter and CMV promoter showed similar activity in MRC-5 cells and MeWo cells, which are susceptible to VZV infection, we next examined whether the HHV-6 MIE promoter could control the expression of foreign genes in VZV.

**Figure 5:**
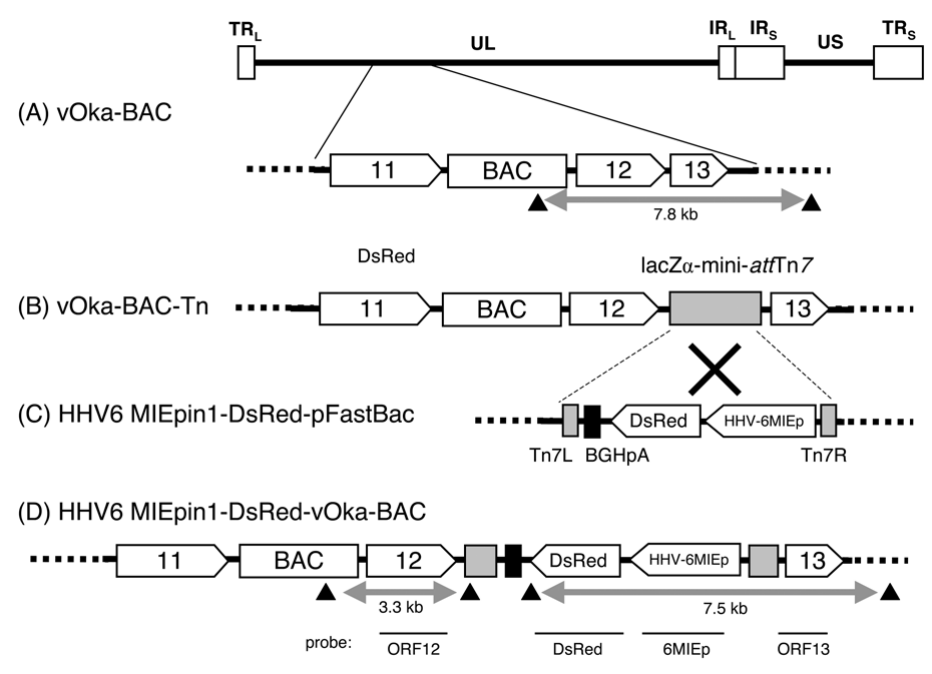
**Construction of the 6MIEpin1-DsRed-vOka genome**. The varicella vaccine Oka strain (vOka)-BAC genome (A) is about 125-kbp long and includes terminal repeats (TRL and TRS), a unique long (UL) domain, internal repeats (IRL and IRS), and a unique short domain (US). The LacZα-mini-attTn7 sequence was inserted between ORF12 and ORF13 of the vOka-BAC genome by RecA-mediated recombination, generating vOka-BAC-Tn (B). The LacZα-mini-attTn7 sequence in the vOka-BAC-Tn genome permitted site-specific insertion of the HHV-6MIEpin1-DsRed-BGH poly(A) signal sequence casette (C) by Tn7-mediated transposition, resulting in the HHV-6MIEp-DsRed-vOka-BAC genome (D). Black arrowheads indicate the *Bam*HI sites. Horizontal bars indicate the region of the probe used for Southern blot analysis.

**Figure 6:**
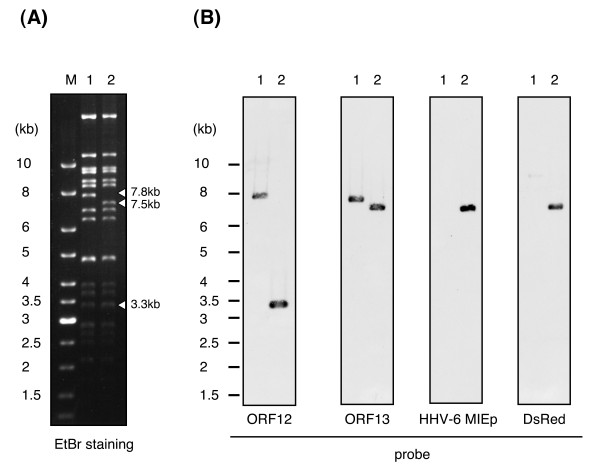
**Confirmation of the insertion of HHV-6MIEp-DsRed into the vOka-BAC genome by Southern blot**. (A) The HHV-6MIEpin1-DsRed-vOka-BAC DNA and the vOka-BAC DNA were digested with *BamHI*, loaded onto a 0.5% agarose gel, and separated by electrophoresis. The DNA fragments were visualized with a UV transilluminator. Arrowheads indicate the band shift following transposition. Each DNA size is shown on the right side of the panel. (B) The blots were hybridized with ORF12, ORF13, DsRed, or HHV-6MIEp probes. Bands were detected by the Enhanced Chemiluminescence (ECL) Direct Nucleic Acid Labeling and Detection System. Lane M: size markers, lane 1: vOka-BAC DNA, lane 2: HHV-6MIEp-DsRed-vOka-BAC DNA.

To investigate this, we incorporated the HHV-6 MIE promoter, with the DsRed2 gene and BGH poly (A) signal sequence, into the VZV vOka BAC genome by Tn7-mediated site-specific transposition (Figure 5). Since the full-length HHV-6 MIE promoter including intron 1 (HHV-6MIEpin1) had the strongest activity of all the promoter variants, we used it for this construct. The DsRed2 gene, which encodes a red fluorescent protein, was used as a reporter gene. The insertion of foreign gene cassette was confirmed by RFLP analysis using *Bam*HI and soutern blot analysis. As shown in Figure 6A, there was a shift in size from 7.8-kbp in the vOka-BAC DNA to 7.5-kbp in the HHV-6MIEpin1-DsRed-vOka-BAC DNA. Furthermore, in the Southern blot analysis, the probes for HHV-6MIEp and DsRed detected bands only in the HHV-6MIEpin1-DsRed-vOka-BAC genome (Figure 6B), indicating that the HHV-6MIEpin1-DsRed cassette had been inserted into the vOka genome.

**Figure 7:**
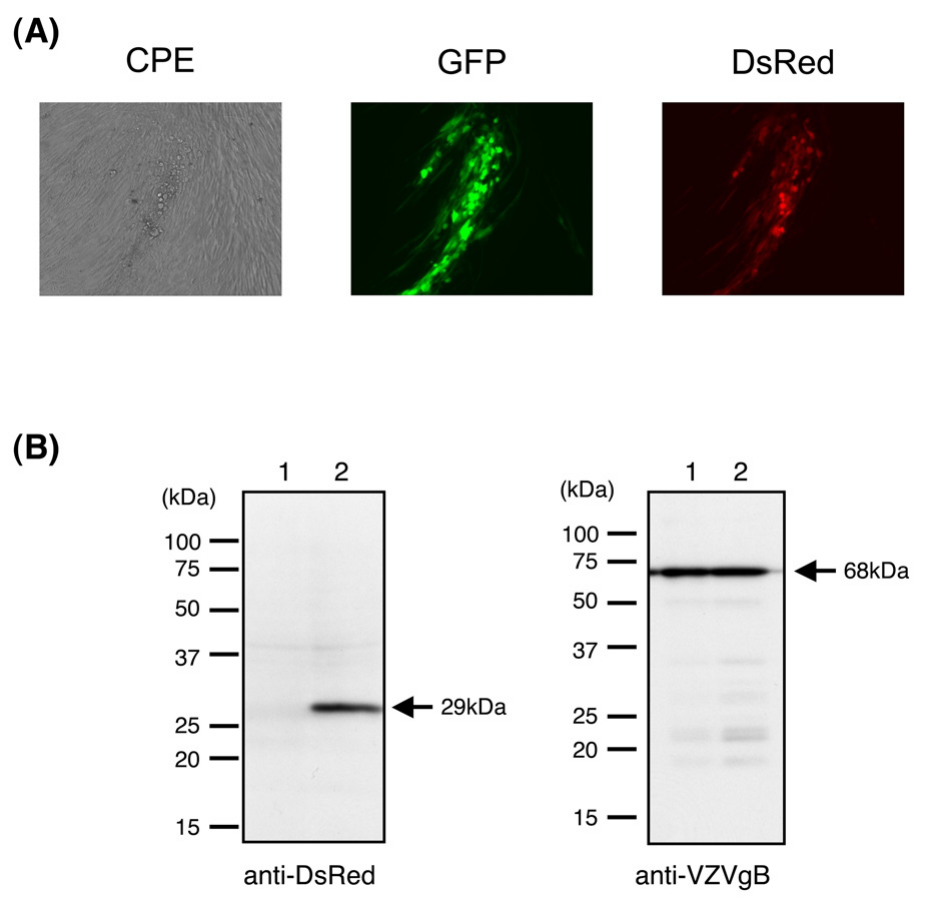
**The expression of heterogous gene under the HHV-6 promoter in recombinant VZV-infected cells**. (A) The HHV-6MIEpin1-DsRed-vOka-BAC DNA was transfected into MRC-5 cells. The infectious virus, reconstituted from the HHV-6MIEp-DsRed-vOka-BAC DNA, caused typical cytopathic effects along with green fluorescence and red fluorescence at 5 days post-transfection. (B) The HHV-6MIEpin1-DsRed-vOka-BAC-infected MRC-5 cells and vOka-BAC-infected MRC-5 cells were lysed in sample buffer, and subjected to Western blot analysis. Blots were reacted with an anti-DsRed mAb or anti-VZV gB Abs. The position and molecular mass in kDa of marker proteins are indicated at the left. Lane 1: vOka-BAC-infected MRC-5 cells, lane 2: HHV-6MIEpin1-DsRed-vOka-BAC-infected MRC-5 cells.

To reconstitute infectious virus from the HHV-6MIEpin1-DsRed-vOka-BAC DNA, MRC-5 cells were transfected with the BAC DNA. Five days after the transfection, typical cytopathic effects (CPEs) were shown. Along with the CPEs, green fluorescence from green fluorescent protein (GFP), which gene was included in BAC sequence, and red fluorescence from DsRed2 were observed by fluorescence microscopy (Figure 7A); this indicated that the HHV-6MIEpin1-DsRed-vOka-BAC had been reconstituted as an infectious recombinant virus expressing DsRed under control of the HHV-6 MIE promoter.

The expression of the DsRed was confirmed by Western blotting analysis (Figure 7B). Recombinant vOka-infected MRC-5 cell lysates were separated by SDS-PAGE and analyzed by Western blotting with an anti-DsRed mAb or anti-VZV gB Ab. The expression of gB, which is a late gene [26], was examined as a positive control of VZV infection. As shown in Figure 7B, the expression of gB was found in lysates from cells infected with either the control rvOka-BAC or HHV-6MIEpin1-DsRed-rvOka-BAC, while the anti-DsRed mAb specifically reacted with a 29-kDa band only in the HHV-6MIEpin1-DsRed-rVoka-BAC-infected cell lysates. These data indicated that the HHV-6 MIE promoter can be used to drive the expression of foreign genes in VZV-infected cells.

## Discussion

The HCMV major immediate early promoter (HCMV MIEp) has been established and used as a tool to drive gene expression by researchers worldwide. HHV-6 also belongs to the beta-herpesviruses, and has a positional homolog of the HCMV MIE gene. As described in the Introduction, HHV-6 replicates and produces progeny in T cells very well; we therefore speculated that the MIE promoter would have stronger promoter activity in T cells than in other cells. Here we identified the region of the HHV-6 major immediate early promoter (HHV-6 MIEp), described in Figure 1. The promoter activity of HHV-6 MIEp was stronger than that of HCMV MIEp in T- cell lines, but not in other adherent cell lines. This feature of HHV-6 MIEp activity is consistent with the fact that HHV-6 is T-cell tropic.

HHV-6 MIEp is predicted to have an NF-κB-binding site. The activity of a mutant HHV-6 MIEp, with the NF-κB-binding site deleted, was dramatically decreased, indicating that the NF-κB-binding site is critical for the promoter activity of HHV-6 MIEp. However, the HCMV MIEp activity was weak compared to that of HHV-6 MIEp in T-cell lines in our study, even though HCMV MIEp also has an NF-κB-binding site that plays a major role in its promoter activity [27,28]. Therefore, another binding site in addition to the NF-κB-binding site might contribute to the T-cell-specific promoter activity of HHV-6 MIEp, or another binding site in HCMV MIEp might have a repressive effect in T cells.

Although the AP-2 and PEA3 binding sites were not found in HHV-6 MIE promoter region by TFSEARCH, R3 region has these binding sites[17,29]. However, in the study of U95 promoter, it has been reported that PEA3 binding sites in R3 region did not bind any proteins[21]. Therefore, PEA3 binding site might have no or low effect on the MIEp activity. The deletion promoter, HHV-6 MIEp-d1, lost two complete AP-2 binding sites and one AP-2 binding site with one nucleotide mutation, compared to full length promoter. Nevertheless, the activity of HHV-6 MIEp-d1 was similar to that of HHV-6 MIEp. Therefore, the AP-2 binding sites might have low effect on the MIEp activitiy.

Adding the first intron (intron 1) of IE1 to HHV-6 MIEp enhanced the promoter activity significantly. When intron 1 was added, the activity of HHV-6 MIEp became markedly greater than that of HCMV in T cells. In adherent cell lines such as MRC-5 and MeWo cells, the activity of HHV-6 MIEp with intron 1 became similar to that of HCMV MIEp. Intron1 of the IE1 region is predicted to have two CCAAT enhancer binding protein (C/EBP) binding sites and an OCT-1-binding site (Figure 3). The transcriptional regulators that bind to these sites might enhance the promoter activity of HHV-6 MIEp. Interestingly, the promoter construct that contained introns 1 and 2 was less active than the promoter containing only intron 1. Further investigation is needed to elucidate the mechanisms involving the intron regions.

We recently developed a recombinant VZV vaccine strain containing the mumps virus HN gene. In this study, we examined whether the HHV-6 MIEp containing intron 1 functioned as a heterologous expression promoter in the VZV vaccine strain. Indeed, in the recombinant VZV, HHV-6 MIEp functioned to drive the expression of the DsRed gene, which is a heterologous gene. These findings indicate that, like the commercially available HCMVp, HHV-6 MIEp is useful for expressing heterologous genes in a VZV vaccine strain.

## Conclusions

Our results show that HHV-6 MIE promoter functions more strongly than CMV MIEp in various T-cell lines. Moreover, the first intron of HHV-6 IE1 gene enhances the promoter activity of HHV-6 MIEp. In addition, the HHV-6 MIEp could drive heterologous gene expression in recombinant varicella-zoster virus-infected cells. These results suggest that HHV-6 MIEp can be used for driving gene expression.

## Methods

### Cells

MRC-5 cells, human lung fibroblasts, were cultured in modified minimum essential medium (MEM) supplemented with 10% fetal bovine serum (FBS). MeWo cells, a human melanoma cell line, and U373 cells, a human astrocytoma cell line, were cultured in Dulbecco's modified Eagle's medium supplemented with 8% FBS. Molt-3 cells, SupT1 cells, and Jurkat cells, which are lymphoblastic T-cell lines, were cultured in RPMI1640 medium supplemented with 8% FBS.

### Plasmids for the luciferase reporter assay

The HHV-6 major immediate-early promoter (HHV-6MIEp) sequence and its deletion mutants were amplified by PCR from the HHV-6B strain HST [30]. The primer sequences are shown in Table 1. The 971-bp fragment located from -983 to -13 bp upstream of exon 1 of IE1, which was amplified using the primer pair 6MIEpF and 6MIEpR, was defined as 6MIEp. The 5' primers named 6MIEpF-732, 6MIEpF-552, 6MIEpF-531, 6MIEpF-381, 6MIEpF-214, 6MIEpF-165, and 6MIEpF-102 were used to generate a series of 5'-deletion mutants. The 3' primers named 6MIEpex2R, 6MIEpex3R, 6MIEpex4R, and 6MIEpex5R were used to amplify HHV-6MIEp including introns 1 to 4, respectively. These amplified fragments were digested and inserted into the pGL3-basic vector (Promega) at the *Hin*dIII and *Xho*I or *Kpn*I site.

**Table 1:** Primers

**Primer**	Sequence*
6MIEpF	5'-tct ctc gag agt taa aga tca gcg ggt ac-3'
6MIEpF-732	5'-agt cgg tac cgg cga atg aga act cta aaa gct c-3'
6MIEpF-552	5'-agt cgg tac cta ctg tgg ttg ggg tct ttc cta c-3'
6MIEpF-531	5'-acc ggt acc tac cca ggc taa cga gaa cc-3'
6MIEpF-381	5'-agt cgg tac cac att cct gtt tca tga tgt gta gc-3'
6MIEpF-214	5'-agt cgg tac ctc ctg ttt ttg agt aag ata tga c-3'
6MIEpF-165	5'-agt cgg tac cag cta att tcc att cca tat ttg tc-3'
6MIEpF-102	5'-agt cgg tac cta cag cga ttg gct cct tca tcc tc-3'
	
6MIEpR	5'-agt cct cga gca ctg aac tgg ctg taa ctt ctg c-3'
6MIEpex2R	5'-tct aag ctt cag caa tcc aat aat tga tg-3'
6MIEpex3R	5'-cat aag ctt gca tac gtt cct cat tgg at-3'
6MIEpex4R	5'-cat aag ctt cca aag ttt tga att ctt ca-3'
6MIEpex5R	5'-cat aag ctt ttt gga tgc aag tgc caa cg-3'
	
DsRed2-HindF	5'-acc aag ctt tac cgg tcg cca cca tgg cct-3'
DsRed2-HindR	5'-acc aag ctt tta tct aga tcc ggt gga tcc-3'
	
ORF12TnFw	5'-tat ctc gag agg tac cgg tga ctt cag ag-3'
ORF12TnRv	5'-cga gga tcc aat caa cca atc aga cct-3'
	
ORF13TnFw	5'-gag gat ccg tac cca caa tat caa gtg gt-3'
ORF13TnRv	5'-gac tcg agc cta ttc gtg tca tct aga tgg-3'

*:underlines indicate restriction enzyme sites.

The CMV MIE promoter sequence was excised with *Nru*I and *Bam*HI from pcDNA3.1(+) (Invitrogen), and inserted into pGL3-basic (Promega) at the *Sma*I and *Bgl*II sites.

The pRL-TK plasmid (Promega), which contains the Renilla luciferase reporter gene under the HSV TK promoter, was used to normalize the transfection efficiency.

### Luciferase reporter assay

Adherent cells (MRC-5, MeWo, and U373) were plated on 24-well plates at a density of 1 × 10^5 ^cells per well on the day before transfection, and were transfected with 1 μg of reporter plasmid and 0.25 μg of pRL-TK plasmid (Promega), using Lipofectamine 2000 (Invitrogen) according to the manufacturer's instructions. Samples containing 4 × 10^5 ^suspended cells (Molt-3, Jurkat, or SupT1) were transfected with 1 μg of reporter plasmid and 0.25 μg of pRL-TK using Lipofectamine2000.

Firefly and Renilla luciferase activities were measured with the Dual-Luciferase Reporter Assay System (Promega) according to the manufacturer's protocol, using a luminometer (Berthold, TriStar LB941). Cells were lysed in 1 × lysis buffer (50 μL/well) for 15 min at room temperature, and each cell lysate was added to a luminometer tube containing 100 μL of assay reagent. The mixture was blended quickly by flicking, and placed in the luminometer for a 1-sec measurement. The transfection efficiency was normalized to the Renilla luciferase activity. The data (mean + SD) were collected from three independent transfections.

### Generation of a recombinant vOka-BAC genome containing HHV-6 MIE promoter

To generate the HHV-6MIEpin1-pFastBac plasmid, the gentamicin-resistance gene and the polyhedrin (PH)-promoter region of the pFastBac1 plasmid (Invitrogen) were replaced with 6MIEp including the intron 1 (HHV-6MIEpin1) sequence.

The DsRed fragment was amplified by PCR using the primer pair DsRed2-HindF and DsRed2-HindR, and *Hin*dIII sites were introduced at both the 5' and 3' ends. The pDsRed2-C1 plasmid (Clontech), in which the *Hin*dIII site had been eliminated, was used as the PCR template. Following amplification, the PCR products were inserted into the HHV-6MIEpin1-pFastBac plasmid at the *Hin*dIII site, generating the HHV-6MIEpin1-DsRed-pFastBac plasmid (Figure 5C). The BGH poly (A) signal sequence was derived from pFastBac plasmid.

The vOka-BAC was obtained using pHA-2 cloning vector (a kind gift from Dr. Ulrich Koszinowski[31]), as described previously[32]. The LacZα-mini-*att*Tn*7 *cassette was inserted into vOka-BAC (Figure 5A) to produce vOka-BAC-Tn (Figure 5B) using RecA-mediated recombination, essentially as described previously [32]. In brief, *E. coli *DH10B electrocompetent cells harboring circular vOka-BAC DNA were co-transformed with 1 μg of the targeting vector, pKO5M-Tn (pKO5M is a kind gift from Dr. Kawaguchi[33]), which contain the LacZα-mini-attTn7 region[33,34], and 3 μg of pDF25(Tet)-- (a kind gift from Dr. J. Heath [35]) by electroporation, using a Gene Pulser II (Bio-Rad, Hercules, CA). The surviving co-integrant colonies, selected by their resistance to chloramphenicol and zeocin, and by a Lac + phenotype on an LB plate containing X-Gal and IPTG, were made electrocompetent and transformed with 1 μg of pDF25(Tet). The *E. coli *DH10B colonies containing the correct survival recombination were then selected by the following criteria: resistance to chloramphenicol, sensitivity to zeocin, and a Lac + phenotype on LB containing X-Gal and IPTG. The insertion of the LacZα-mini-attTn7 sequence into the BAC genome was confirmed by PCR and Southern blotting (Data not shown).

The HHV-6MIEpin1-DsRed cassette was inserted into the vOka-BAC-Tn genome using Tn7-mediated site-specific transposition, essentially as described previously [34]. In brief, *E. coli *DH10B harboring the vOka-BAC-Tn genome was transformed with HHV-6MIEpin1-DsRed-pFastBac and pMON7124 (Invitrogen), a helper plasmid for transposition. The pMON7124 plasmid DNA was isolated from DH10Bac cells (Invitrogen). The transformed *E. coli *was cultured on LB containing X-gal and IPTG for blue/white selection. The white colonies were analyzed by PCR to verify the insertion of the DsRed expression cassette (data not shown). This completed the construction of the HHV-6MIEpin1-DsRed-vOka-BAC genome (Figure 5D).

### Southern blot analysis

The HHV-6MIEpin1-DsRed-vOka-BAC DNA was extracted using a NucleoBond BAC 100 kit (Macherey-Nagel) following the manufacturer's instructions.

The BAC DNA was then digested with *Bam*HI, loaded onto a 0.5% agarose gel, and separated by electrophoresis at 20 V for 72 hrs. The DNA fragments were visualized with a UV transilluminator and then transferred onto a nylon membrane (Hybond-N+) (GE Healthcare Bio-sciences). The blots were hybridized with ORF12, ORF13, DsRed, or HHV-6MIEp probes labeled with horseradish peroxidase. These probes were amplified by PCR using the following primer pairs: ORF12TnFw/ORF12TnRv, ORF13TnFw/ORF13TnRv, DsRed-HindF/DsRed-HindR, and 6MIEpF-552/6MIEpex2R, respectively (the primer sequences are shown in Table 1). Bands were detected by the Enhanced Chemiluminescence (ECL) Direct Nucleic Acid Labeling and Detection System (GE Healthcare Bio-sciences) following the manufacturer's instructions.

### Reconstitution of infectious virus from vOka-BAC DNA

Reconstitution of the recombinant virus, named HHV-6MIEpin1-DsRed-rvOka, was performed as described previously [32,36]. Briefly, MRC-5 cells were transfected with 1 μg of HHV-6MIEpin1-DsRed-vOka-BAC DNA by electroporation, using a Nucleofection unit (Amaxa Biosystems). The transfected cells were then cultured in MEM supplemented with 3% FBS for 3-5 days, and were observed under a microscope until a typical cytopathic effect with green and red fluorescence appeared.

### Western blot analysis

The HHV-6MIEp-DsRed-vOka-BAC-infected MRC-5 cells were lysed in sample buffer [32 mM Tris-HCl (pH 6.8), 1.5% SDS, 5% glycerol, 2.5% 2-mercaptoethanol], separated by SDS-polyacrylamide gel electrophoresis (PAGE), and electrotransferred onto PVDF membranes (Bio-Rad Laboratories). A monoclonal antibody (mAb) against DsRed (Clontech) was purchased, and an anti-VZV gB monospecific antibody (Ab) was produced in our laboratory [26]. Blots were blocked with blocking buffer (PBS, 5% skim milk, 0.1% Tween-20) and reacted with the anti-DsRed mAb or anti-gB Ab in blocking buffer. The protein bands were developed with horseradish peroxidase-conjugated secondary antibodies (GE Healthcare) and ECL detection reagents (GE Healthcare Bio-Sciences), following the manufacturer's instructions.

## Competing interests

The authors declare that they have no competing interests.

## Authors' contributions

MM performed and analyzed the experiments, and drafted the manuscript. TM participated in the design of the study partly and performed the experiments partly. KY analyzed the study. YM participated in its design and coordination, analyzed the study, and drafted the manuscript. All authors read and approved the final manuscript.
